# Tetrandrine enhances the ubiquitination and degradation of Syk through an AhR-c-src-c-Cbl pathway and consequently inhibits osteoclastogenesis and bone destruction in arthritis

**DOI:** 10.1038/s41419-018-1286-2

**Published:** 2019-01-15

**Authors:** Yugai Jia, Yu Tao, Changjun Lv, Yufeng Xia, Zhifeng Wei, Yue Dai

**Affiliations:** 1 Department of Pharmacology of Chinese Materia Medica, School of Traditional Chinese Pharmacy, China Pharmaceutical University, 24 Tong Jia Xiang, Nanjing, 210009 China; 2 Department of Pharmacology, Hebei University of Chinese Medicine, No. 326 South Xinshi Road, Shijiazhuang, 050091 Hebei China

## Abstract

Recently, we reported that tetrandrine, a natural alkaloid, could inhibit the osteoclastogenesis and bone erosion through enhancing the ubiquitination and degradation of spleen tyrosine kinase (Syk). Herein, we addressed whether and how aryl hydrocarbon receptor (AhR) mediate the effect of tetrandrine. In vitro, tetrandrine was shown to repress RANKL-induced osteoclastogenesis and the expression of osteoclast-related marker genes, which was almost completely reversed by either AhR antagonist CH223191 or siRNA. In pre-osteoclasts, tetrandrine enhanced the ubiquitination and degradation of Syk through the AhR/c-src/c-Cbl signaling pathway, downregulated the expression of phospho-Syk and phospho-PLCγ2, and inhibited the nuclear translocation of NFATc1, a master transcription factor for osteoclastogenesis. Notably, tetrandrine acted through the non-genomic pathway of the ligand-activated AhR, as evidenced by the fact that the effect of tetrandrine did not change in the absence of AhR nuclear translocator. In collagen-induced arthritis rats, oral administration of tetrandrine decreased the number of phospho-Syk-positive cells and osteoclasts, and reduced the bone erosion in the areas of the proximal tibial epiphysis excluding the cortical bone. A combined use with CH223191 almost abolished the effect of tetrandrine. These findings revealed that tetrandrine enhanced the ubiquitination and degradation of Syk and consequently repressed the osteoclastogenesis and bone destruction through the AhR-c-src-c-Cbl pathway.

## Introduction

The maintenance of bone mineral homeostasis is dependent on the balance of osteoclastic bone resorption and osteoblastic bone formation. An abnormal bone resorption would result in bone disorders such as osteoporosis and rheumatoid arthritis (RA), which are characterized by the excessive bone loss and absolute increase in the number of osteoclasts^1,2^. Therefore, targeting the differentiation and function of osteoclasts would be beneficial for the treatment of the bone loss disorders. Osteoclasts are large multinucleated cells derived from monocyte/macrophage lisneage cells of hematopoietic cells. Osteoclastogenesis is principally triggered by receptor activator of nuclear factor κB ligand (RANKL) via its receptor RANK. RANKL-RANK interaction leads to the recruitment of TNF receptor-associated factor 6 (TRAF6), which activates MAPKs, AP-1, NF-κB and other downstream molecules crucial for the onset of osteoclastogenesis^3^. Moreover, RANKL-driven osteoclastogenesis might depend on the generation of a calcium signal through the activation of spleen tyrosine kinase (Syk) and phospholipase-Cγ (PLCγ). The calcium signal leads to the activation of nuclear factor of activated T cells c1 (NFATc1), which can directly regulate the expression of osteoclast-related marker genes, such as tartrate resistant acid phosphatase (TRAP) and dendritic cell-specific transmembrane protein (DC-STAMP). Specific down-regulation of the above signaling pathways substantially contributes to the repression of osteoclastogenesis^4^.

Aryl hydrocarbon receptor (AhR) is a ligand-activated transcription factor that regulates the xenobiotic metabolism. Inactive AhR localizes in the cytoplasm forming a multi-protein complex with other proteins such as HSP90, XAP2 and c-src. Upon ligand binding, AhR dissociates from this complex, and translocates into the nucleus where it forms a heterodimer with its partner AhR nuclear translocator (Arnt). Ultimately, AhR binds to the dioxin response element of the promoter region of its target genes such as CYP1A1, and transcriptionally activates them^5^. Increasing evidence indicates that AhR regulates osteoclastogenesis in a ligand-, species- and concentration-specific manner. Tetrachlorodibenzo-p-dioxin (TCDD), benzo[a]pyrene (Bap) and 3, 3’-diindolylmetheane (DIM), the potent AhR agonists, could inhibit osteoclastogenesis and increase trabecular bone volume (BVF) and mineral density (BMD) in mice. Unfortunately, most of the classical AhR ligands have significant toxicities, which limit their use as therapeutic agents in animals or humans^6–8^. There is a need to search and develop safe AhR ligands as therapeutic agents of osteoclastogenesis-related diseases.

Tetrandrine is a bisbenzylisoquinoline alkaloid. Accumulative evidence obtained from clinical and animal trials indicates that tetrandrine is relatively safer, and has been used to treat rheumatalgia and arthralgia in China for decades^9^. Our previous studies showed that tetrandrine could inhibit RANKL-induced osteoclastogenesis via enhancing the ubiquitylation and degradation of Syk, and attenuate the bone destruction in collagen-induced arthritis (CIA) rats^10^. Furthermore, tetrandrine was found to be a potential ligand of AhR, which could activate AhR and increase the expression of the downstream target gene CYP1A1 in murine T cells^9^. The objective of the present study was to explore whether and how AhR mediate the effects of tetrandrine and DIM on the osteoclastogenesis and bone destruction.

## Results

### Tetrandrine and DIM inhibited osteoclastogenesis in an AhR-dependent manner

**Fig. 1:**
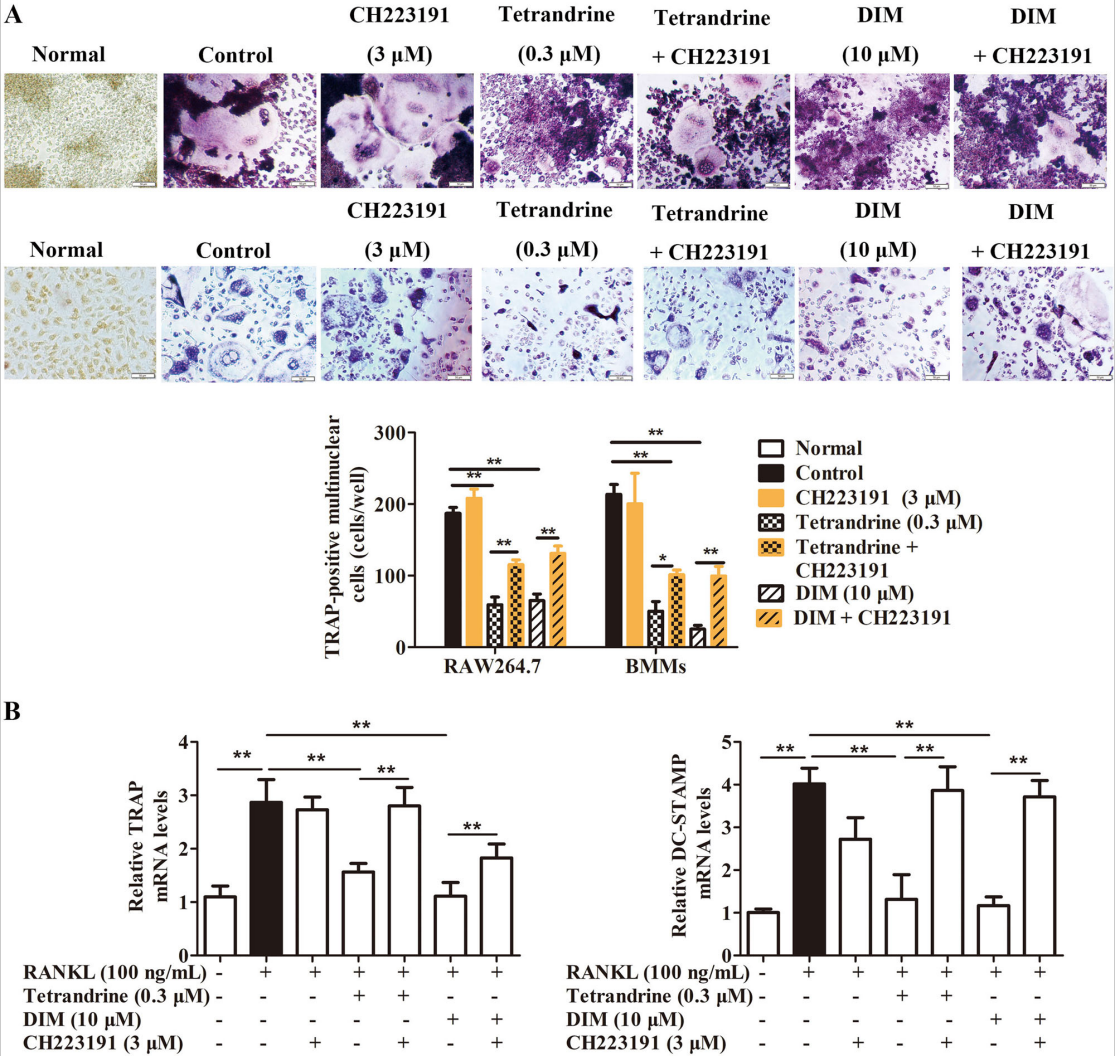
Tetrandrine and DIM inhibited the osteoclastogenesis in an AhR-dependent manner. **a** RAW264.7 cells (up) and BMMs (down) were treated with indicated compounds in the presence or absence of RANKL (100 ng/mL) for 5 days. The osteoclasts were stained using a TRAP kit according to the manufacture’s protocol. TRAP-positive multinucleated cells (nuclei ≥ 3) were counted using an inverted microscope. **b** BMMs were treated with indicated compounds in the presence or absence of RANKL (100 ng/mL) for 24 h. The cells were harvested and lysed, and the mRNA expressions of TRAP and DC-STAMP were detected by quantitative PCR. The results are representative of three independent experiments. ^*^P *<* 0.05, ^**^P *<* 0.01 vs. indicated group

The test concentration of tetrandrine was chosen according to our previous findings in which 0.3 μM of tetrandrine could significantly inhibit the osteoclastogenesis with no evident cytotoxicity^10^. To explore whether tetrandrine and DIM regulate the osteoclastogenesis through the AhR pathway, pre-osteoclasts RAW264.7 cells and BMMs were treated with tetrandrine and DIM in the presence of or absence of CH223191 and AhR siRNA under osteoclast-differentiation conditions. The results showed that tetrandrine (0.3 μM) and DIM (10 μM) markedly inhibited the osteoclastogenesis (Fig. 1a and Supplementary Figure 1A) as well as the expression of the osteoclast marker genes TRAP and cathepsin K (Fig. 1b and Supplementary Figure 1B). CH223191 or AhR siRNA markedly diminished the inhibitory effects of tetrandrine and DIM. The findings suggested that tetrandrine and DIM inhibited the osteoclastogenesis in an AhR-dependent manner.

### Tetrandrine and DIM could activate AhR in the pre-osteoclasts

**Fig. 2:**
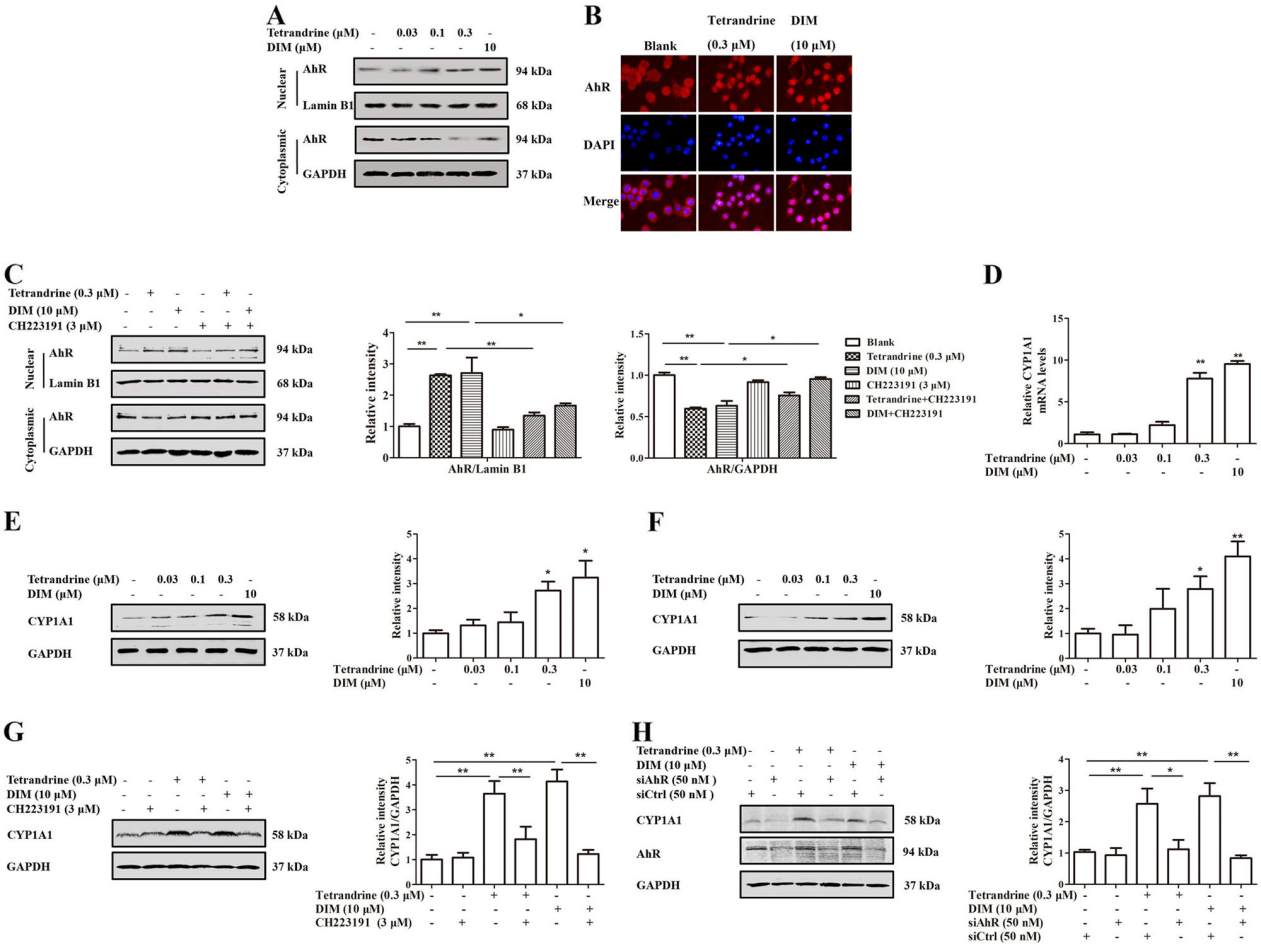
Tetrandrine and DIM activated AhR in the pre-osteoclasts. RAW264.7 cells or BMMs were treated with tetrandrine or DIM for 6 h. **a** The nuclear translocation of AhR in RAW264.7 cells were analyzed by western blots. **b** The nuclear localization of AhR in BMMs was visualized by immunofluorescence analysis. **c** The nuclear translocation of AhR in BMMs was analyzed by western blots. **d** The CYP1A1 mRNA expression in RAW264.7 cells was detected using the quantitative PCR assay. **e**, **f** CYP1A1 protein expression in RAW264.7 cells and BMMs was detected using western blots assay. **g** The level of CYP1A1 in RAW264.7 cells was evaluated by western blots. **h** The expression of CYP1A1 in RAW264.7 cells was analyzed using western blots. Results are expressed as the means ± S.E.M. from at least 3 independent experiments. *P < 0.05, **P *<* 0.01 vs. indicated group. siAhR: AhR siRNA. siCtrl: control siRNA

To verify whether tetrandrine and DIM could activate AhR in the pre-osteoclasts, the effects of tetrandrine and DIM on AhR nuclear import and the expression of CYP1A1 in RAW264.7 cells/BMMs were investigated. Figure 2a, b showed that tetrandrine and DIM enhanced the nuclear translocation of AhR. While, CH223191 almost abolished the effects of tetrandrine and DIM on the nuclear translocation of AhR (Fig. 2c). Consistent with this, tetrandrine and DIM enhanced the expression of CYP1A1 at the protein and mRNA levels, suggesting that tetrandrine and DIM could activate AhR in the pre-osteoclasts (Fig. 2d–f). Furthermore, CH223191 or AhR siRNA abolished the tetrandrine- or DIM-induced CYP1A1 expression (Fig. 2g, h), suggesting that AhR was required for the expression of CYP1A1 induced by tetrandrine or DIM in the pre-osteoclasts.

### Tetrandrine and DIM enhanced the ubiquitylation and degradation of Syk in an AhR-dependent manner

**Fig. 3:**
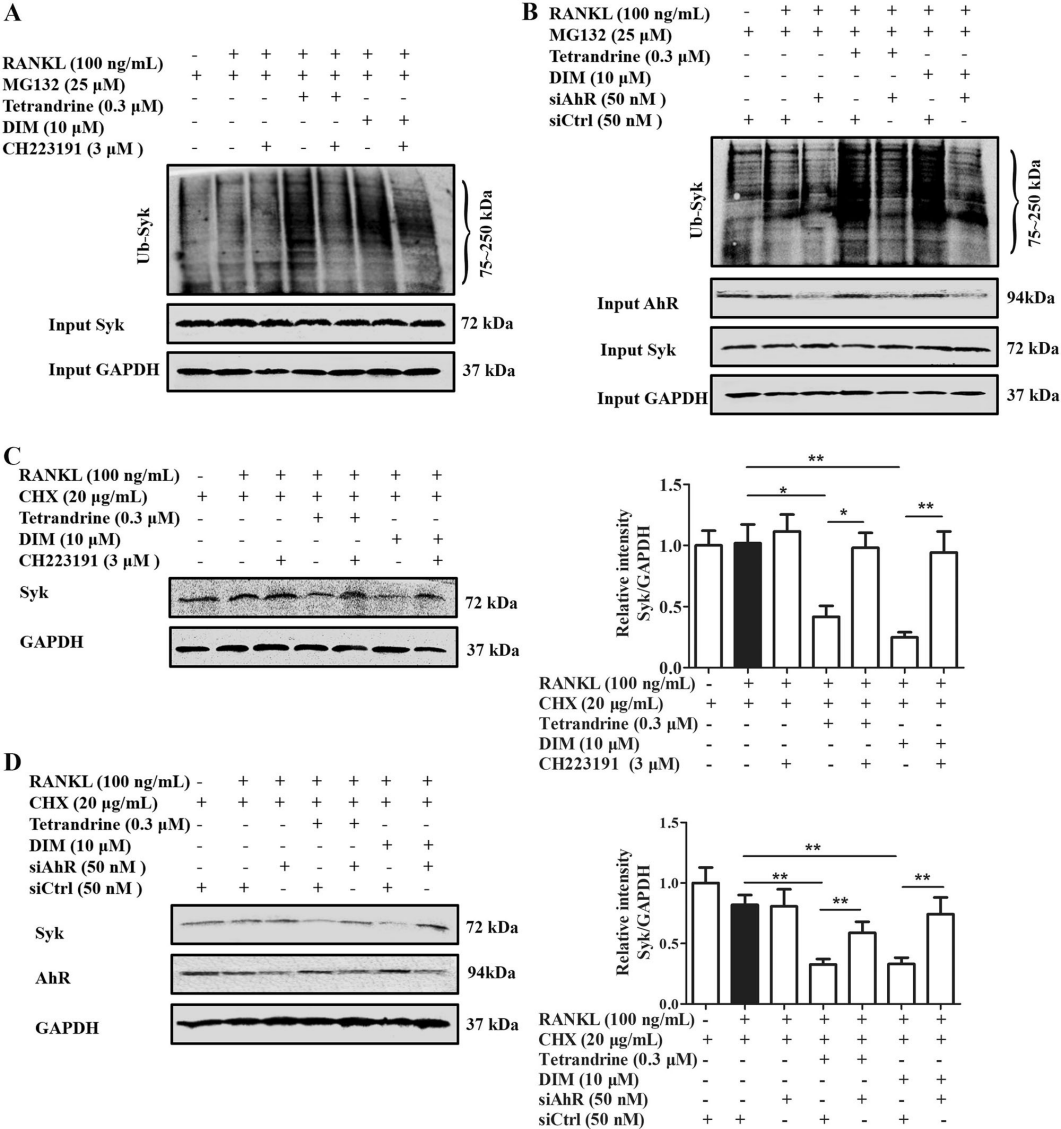
Tetrandrine and DIM enhanced the ubiquitylation and degradation of Syk in an AhR-depend manner. **a**, **b** RAW264.7 cells were treated with tetrandrine (0.3 μM) or DIM (10 μM) in the presence or absence of CH223191 (3 μM) or siAhR for 6 h. The cells were treated with MG132 (25 μM) for 2 h, and then exposed to RANKL (100 ng/mL) for 15 min. The cells lysates were immunoprecipitated with antibody for Syk, and the precipitated Syk was detected by western blots with special antibody against ubiquitin. **c**, **d** RAW264.7 cells were treated with tetrandrine (0.3 μM) or DIM (10 μM) in the presence or absence of CH223191 (3 μM) or siAhR for 6 h. The cells were treated with CHX (20 μg/mL) for 2 h, and then exposed to RANKL (100 ng/mL) for 2 h. The cell lysates were analyzed using western blots with antibodies against Syk and GAPDH. Results are expressed as the means ± S.E.M. from at least 3 independent experiments. *P < 0.05, **P < 0.01 vs. indicated group. siAhR: AhR siRNA. siCtrl: control siRNA

There was a report indicating that AhR could function as an E3 ubiquitin ligase to induce the ubiquitination and degradation of the target substrates by proteasome^11^. We wondered that AhR might mediate the tetrandrine- or DIM-induced the ubiquitination and degradation of Syk during osteoclastogenesis. To test this hypothesis, we used CH223191 and AhR siRNA in combination with tetrandrine and DIM under osteoclast-differentiation conditions. The results showed that tetrandirne and DIM themselves significantly enhanced the ubiquitination and degradation of Syk induced by RANKL in the pre-osteoclasts (Fig. 3a, b), which could be largely diminished by either CH223191 or AhR siRNA (Fig. 3c, d), suggesting that the activation of AhR substantially contributed to the ubiquitination and degradation of Syk induced by tetrandrine or DIM.

### Tetrandrine and DIM enhanced the degradation of Syk through the non-genomic route of AhR

**Fig. 4:**
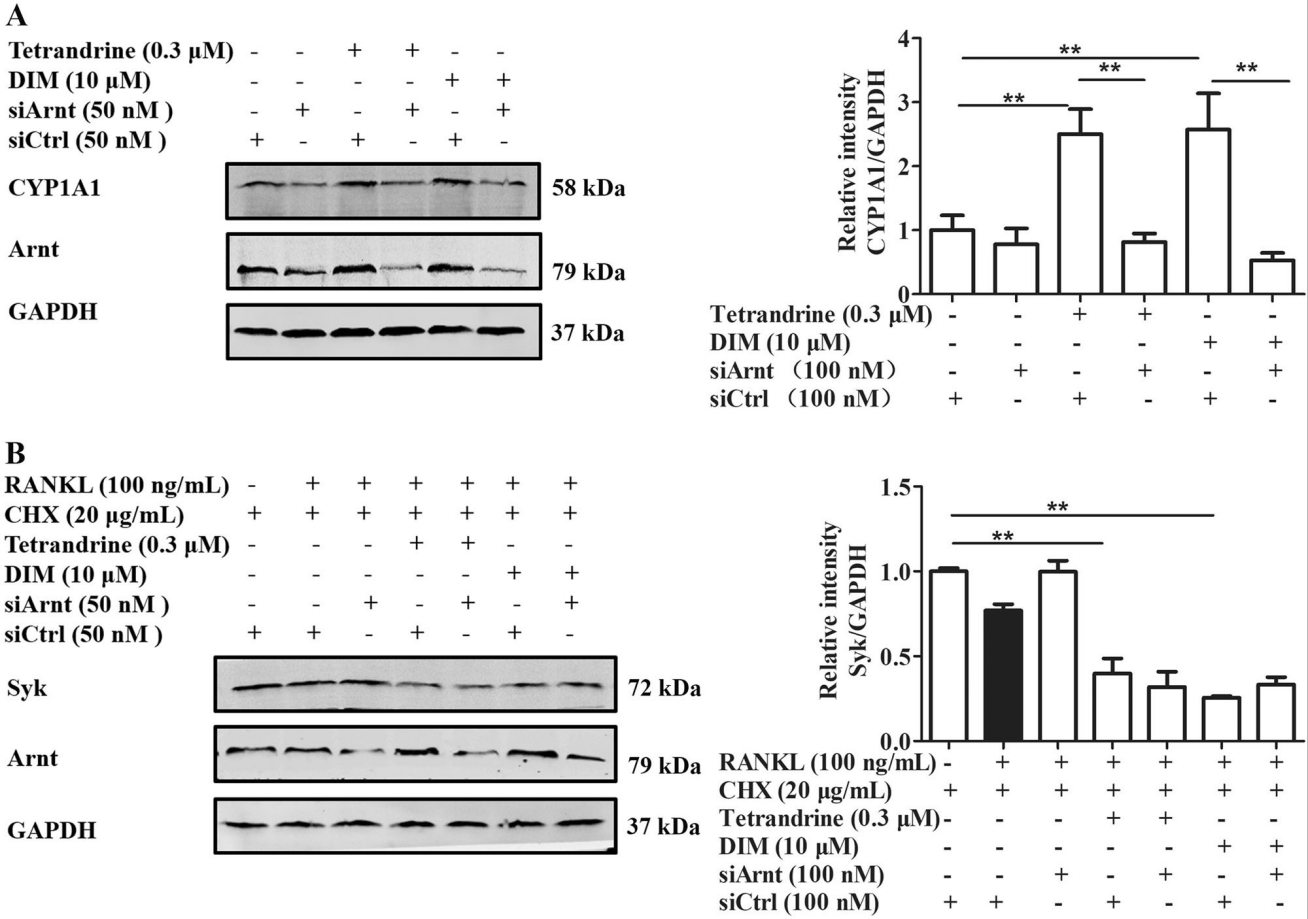
Tetrandrine and DIM enhanced the degradation of Syk though the non-genomic route of AhR. RAW264.7 cells were transfected with either siArnt or control siCtrl, and then treated with tetrandrine or DIM for 6 h. **a** CYP1A1 protein expression was detected using western blots assay. **b** The cells were treated with CHX (20 μg/mL) for 2 h, and exposed to RANKL (100 ng/mL) for 2 h. The cell lysates were analyzed using western blots with antibodies against Syk or GAPDH. Results are expressed as the means ± S.E.M. from at least 3 independent experiments. *P < 0.05, **P < 0.01 *vs*. indicated group. siAhR: AhR siRNA. siCtrl: control siRNA

The ligands of AhR might function through the classical genomic route or the alternative non-genomic route^12^. To recognize the action route of tetrandrine and DIM after activating AhR, we used Arnt siRNA to block the genomic route in the pre-osteoclasts. Figure 4a showed that Arnt siRNA transfection almost completely abolished the enhancement of tetrandrine and DIM on the expression of CYP1A1, but did not affect the promotion on the Syk degradation (Fig. 4b). It was implied that tetrandrine and DIM enhanced the degradation of Syk in the pre-osteoclasts through the non-genomic route of AhR, independent of the expression of CYP1A1.

### Tetrandrine and DIM enhanced the degradation of Syk through the activation of c-Cbl

**Fig. 5:**
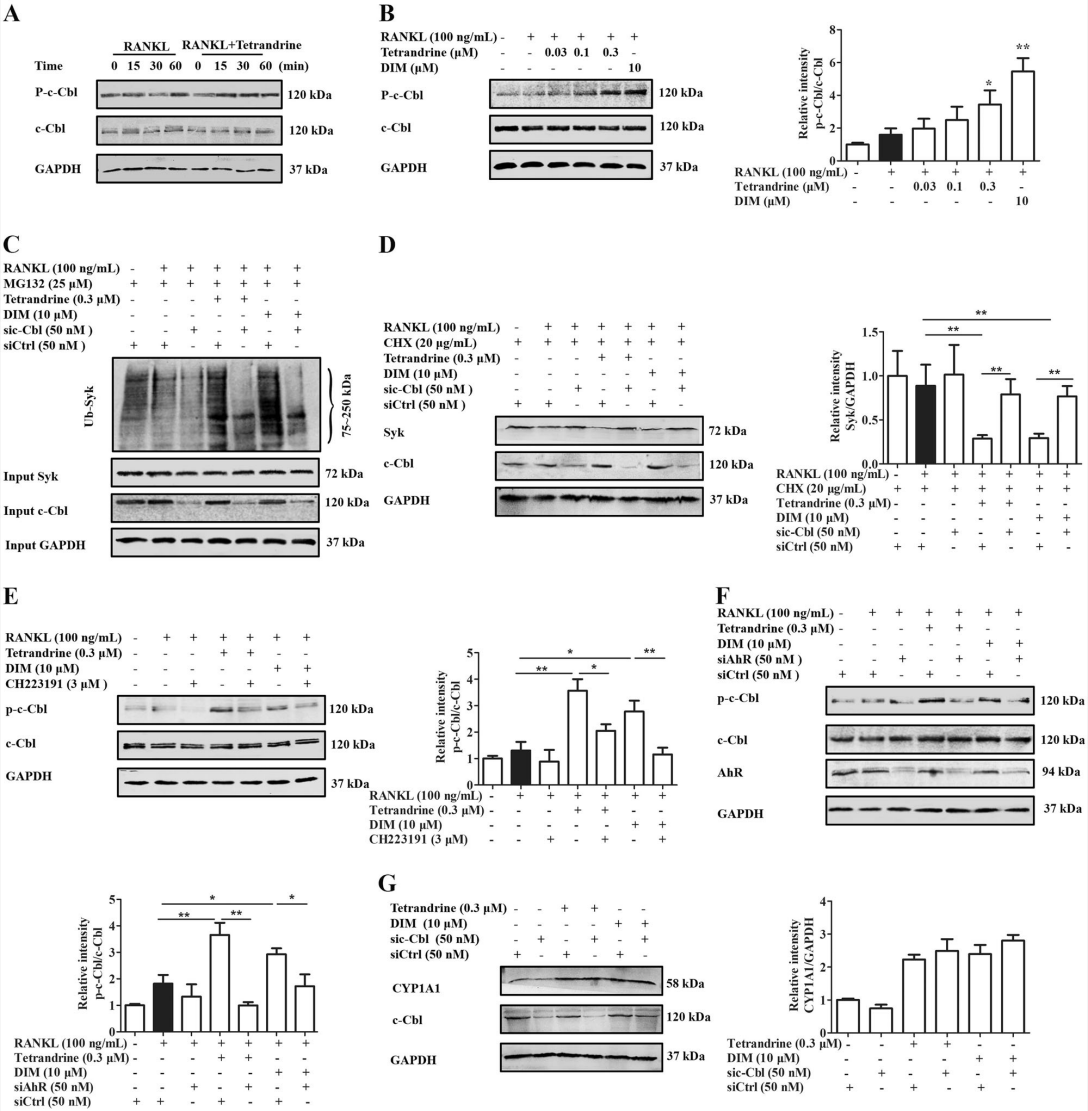
Tetrandrine and DIM enhanced the ubiquitination and degradation of Syk through the activation of c-Cbl. **a** RAW264.7 cells were treated with or without tetrandrine (0.3 μM) for 6 h, and followed with RANKL (100 ng/mL) for indicated periods. The levels of p-c-Cbl and c-Cbl were evaluated by western blots. **b** RAW264.7 cells were treated with tetrandrine (0.3 μM) or DIM (10 μM) for 6 h, and followed with RANKL (100 ng/mL) for 15 min. The levels of p-c-Cbl and c-Cbl were evaluated by western blots. **c** RAW264.7 cells were transfected with either sic-Cbl or siCtrl, and followed by treatment with tetrandrine (0.3 μM) or DIM (10 μM) for 6 h. The cells were treated with MG132 (25 μM) for 2 h, and exposed to RANKL (100 ng/mL) for 15 min. The cells lysates were immunoprecipitated with an antibody against Syk, and precipitated Syk was detected by western blots with special antibody against ubiquitin. **d** RAW264.7 cells were transfected with either sic-Cbl or siCtrl, and followed by treatment with tetrandrine (0.3 μM) or DIM (10 μM) for 6 h. The cells were treated with CHX (20 μg/mL) for 2 h, and exposed to RANKL (100 ng/mL) for 2 h. The cell lysates were analyzed using western blots with antibodies against Syk and GAPDH. **e**, **f** RAW264.7 cells were treated with tetrandrine (0.3 μM) or DIM (10 μM) for 6 h in the presence or absence of CH223191 (3 μM) or siAhR, and followed with RANKL (100 ng/mL) for 15 min. The levels of p-c-Cbl and c-Cbl were evaluated by western blots. (**g**) RAW264.7 cells were transfected with either sic-Cbl or siCtrl, and followed by treatment with tetrandrine (0.3 μM) or DIM (10 μM) for 6 h. The levels of CYP1A1 and GAPDH were evaluated by western blots. Results are expressed as the means ± S.E.M. from at least 3 independent experiments. *P < 0.05, **P *<* 0.01 vs. indicated group. siAhR: AhR siRNA. siCtrl: control siRNA. sic-Cbl: c-Cbl siRNA

Syk activation terminates in its ubiquitinated degradation, and E3 ubiquitin ligase c-Cbl is a necessary factor for this process^13^. Herein, we explored the participation and role of c-Cbl in the tetrandrine- and DIM-induced ubiquitination of Syk under the osteoclast differentiation conditions. Figure 5a, b showed that tetrandrine and DIM markedly increased the level of p-c-Cbl in the pre-osteoclasts after treatment for 15 min. The c-Cbl knockdown by siRNA abolished the tetrandrine- or DIM-enhanced ubiquitylation and degradation of Syk (Fig. 5c, d), suggesting that c-Cbl played a critical role in the ubiquitylation and degradation of Syk induced by tetrandrine or DIM. Furthermore, Fig. 5e, f showed that CH223191 and AhR siRNA markedly reversed the enhancement of tetrandrine and DIM on the phosophorylation of c-Cbl in the pre-osteoclasts. While, c-Cbl siRNA did not affect the expression of CYP1A1 induced by tetrandrine or DIM (Fig. 5g). These results indicated that tetrandrine and DIM enhanced the degradation of Syk through the activation of c-Cbl via AhR.

### Tetrandrine and DIM promoted the activation of c-Cbl through the AhR-c-src pathway

**Fig. 6:**
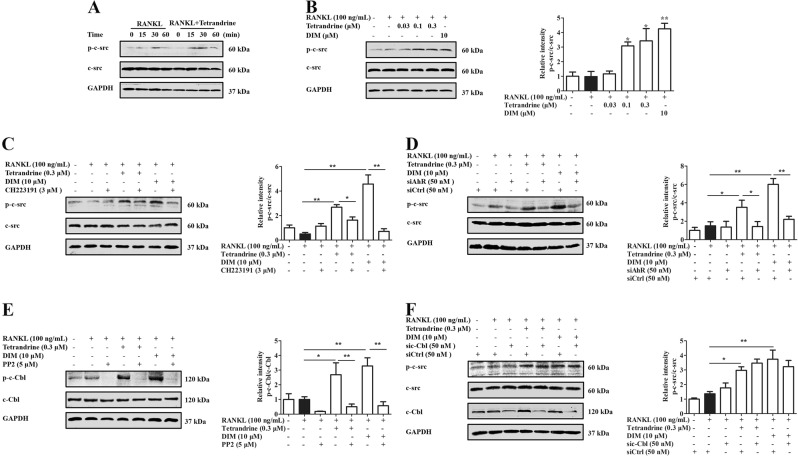
Tetrandrine and DIM enhanced the activation of c-Cbl through the AhR-c-src pathway. **a** RAW264.7 cells were treated with or without tetrandrine (0.3 μM) for 6 h, and then treated with RANKL (100 ng/mL) for indicated periods. The levels of p-c-src and c-src were evaluated by western blots. **b** RAW264.7 cells were treated with tetrandrine (0.3 μM) or DIM (10 μM) for 6 h, and followed with RANKL (100 ng/mL) for 15 min. The levels of p-c-src and c-src were evaluated by western blots. **c**, **d** RAW264.7 cells were treated with tetrandrine (0.3 μM) or DIM (10 μM) for 6 h in the presence or absence of CH223191 (3 μM) or siAhR, and followed with RANKL (100 ng/mL) for 15 min. The levels of p-c-src and c-src were evaluated by western blots. **e** RAW264.7 cells were treated with tetrandrine (0.3 μM) or DIM (10 μM) for 6 h in the presence or absence PP2 (5 μM), and followed with RANKL (100 ng/mL) for 15 min. The levels of p-c-Cbl and c-Cbl were evaluated by western blots. **f** RAW264.7 cells were transfected with either sic-Cbl or siCtrl, and followed by treatment with tetrandrine (0.3 μM) or DIM (10 μM) for 6 h, and then exposed to RANKL (100 ng/mL) for 15 min. The levels of p-c-Cbl and c-Cbl were evaluated by western blots. Results are expressed as the means ± S.E.M. from at least 3 independent experiments. *P < 0.05, **P < 0.01 vs. indicated group. siAhR: AhR siRNA. siCtrl: control siRNA. sic-Cbl: c-Cbl siRNA

There were reports indicating that the activation of c-Cbl could be induced by the non-receptor tyrosine kinase c-src, a chaperonin of AhR^14–16^. We therefore examined whether c-src played a key role in the enhancement of tetrandrine and DIM on AhR-mediated activation of c-Cbl under the osteoclast differentiation conditions. As shown in Fig. 6a, b, tetrandrine and DIM significantly enhanced the phosphorylation of c-src in the pre-osteoclasts. CH223191 or AhR siRNA largely diminished the enhancement effects of tetrandrine and DIM (Fig. 6c, d), suggesting that tetrandrine and DIM promoted the phosphorylation of c-src *via* AhR. Moreover, PP2, a specific inhibitor of c-src, almost completely eliminated the phosphorylation of c-Cbl induced by tetrandrine or DIM (Fig. 6e). While, c-Cbl siRNA did not affect the enhancement of tetrandrine and DIM on the activation of c-src (Fig. 6f). These results indicated that c-Cbl might be the downstream effector of c-src in the pre-osteoclasts.

### Tetrandrine and DIM enhanced the dissociation of AhR from AhR-c-src complex, and the combination with c-Cbl and Syk

To further recognize the relationship among AhR, c-src, c-Cbl and Syk, co-immunoprecipitation assays were performed to examine the effect of tetrandrine and DIM on the association of these proteins. As shown in Supplementary Figure 2, tetrandrine and DIM enhanced the dissociation of AhR from c-src, and the binding of AhR to c-Cbl and Syk in the pre-osteoclasts.

### Tetrandrine and DIM inhibited the activation of Syk-PLCγ2 signaling pathway through AhR

**Fig. 7:**
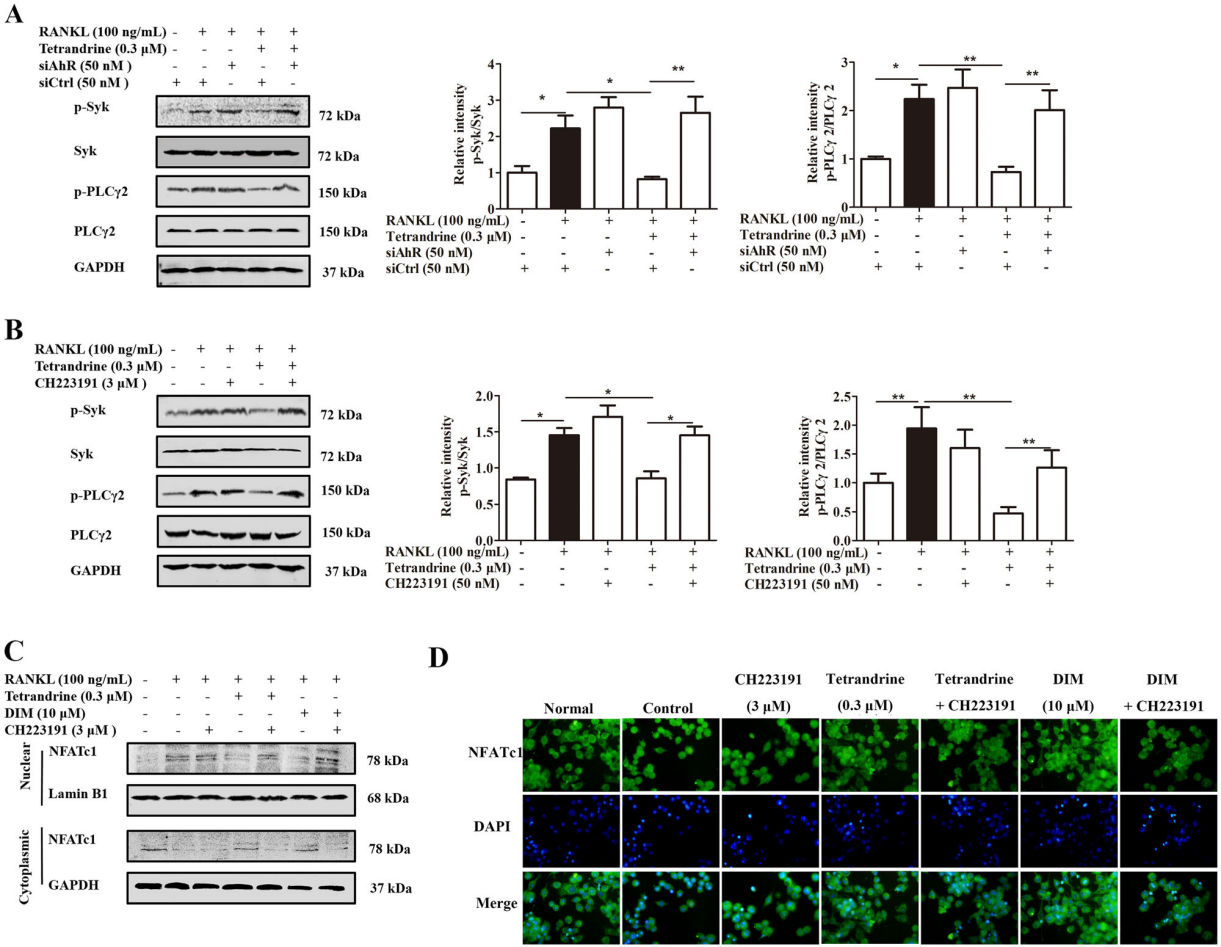
Tetrandrine and DIM inhibited the activation of Syk-PLCγ2 signaling pathway through AhR. **a** RAW264.7 cells were transfected with either siAhR, and followed by treatment with tetrandrine (0.3 μM) for 6 h, and then exposed to RANKL (100 ng/mL) for 15 min. The levels of p-Syk, Syk, p-PLCγ2 and PLCγ2 were evaluated by western blots. **b** BMMs were treated with tetrandrine (0.3 μM) for 6 h in the presence or absence of CH223191 (3 μM), and followed with RANKL (100 ng/mL) for 15 min. The levels of p-Syk, Syk, p-PLCγ2 and PLCγ2 were evaluated by western blots. **c**, **d** BMMs were treated with tetrandrine (0.3 μM) or DIM (10 μM) for 6 h in the presence or absence of CH223191 (3 μM), and followed with RANKL (100 ng/mL) for 40 min. The localization of NFATc1 was visualized by western blots and immunofluorescence analysis. Results are expressed as the means ± S.E.M. from at least 3 independent experiments. *P < 0.05, **P < 0.01 *vs*. indicated group. siAhR: AhR siRNA. siCtrl: control siRNA

The ubiquitination and degradation of Syk should result in the reduction of the level of p-Syk^13,17^. To define whether tetrandrine and DIM inhibited the activation of Syk signaling pathway through AhR, we treated pre-osteoclasts with CH223191 or AhR siRNA in the presence or absence of tetrandrine under the osteoclast-polarizing condition. As shown in Fig. 7a, b, CH223191 and AhR siRNA themselves did not show obvious effect on the activation of Syk and PLCγ2 induced by RANKL, but significantly diminished the inhibitory effect of tetrandrine. Consistently, immunofluorescence and western blots assays showed that the inhibition of tetrandrine and DIM on the nuclear translocation of NFATc1 was also diminished by CH223191 treatment (Fig. 7c, d).

### Tetrandrine and DIM inhibited the osteoclastogenesis and bone destruction in rats with collagen-induced arthritis through the AhR pathway

**Fig. 8:**
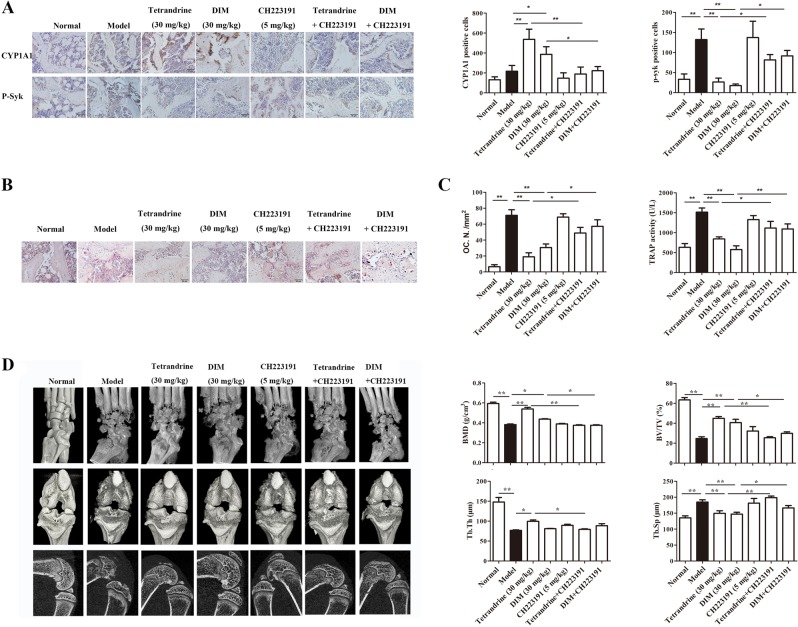
Tetrandrine and DIM inhibited the osteoclastogenesis and bone destruction in rats with collagen-induced arthritis through the AhR pathway. **a**The numbers of CYP1A1- and p-Syk-positive cells were assessed by immunohistochemical staining from the paraffin-embedded proximal tibial epiphysis excluding the cortical bone sections (*n* = 6). **b** The tibia sections were stained with TRAP, and the numbers of osteoclasts were determined (*n* = 6). **c** The serum levels of the TRAP5b in each group were analyzed by ELISA kit (*n* = 6). **c** Micro-CT scan was performed to show the three-dimensional reconstructed bones of ankle and knee joints and the two-dimensional reconstructed bones of knee joints in different groups (*n* = 6). **d** The BMD, BV/TV, Tb.Th and Tb.Sp values of the proximal tibial epiphysis excluding the cortical bone were analyzed using a CTAn software (*n* = 6). Results are expressed as the means ± S.E.M.. *P < 0.05, **P < 0.01 vs. indicated group

To further ascertain the causal link between AhR activation, Syk activation, osteoclastogenesis and bone destruction, tetrandrine and DIM were orally administered in combination with CH223191 in collagen-induced arthritis (CIA) rats. As shown in Fig. 8, tetrandrine and DIM markedly increased the number of CYP1A1-positive cells, decreased the number of p-Syk positive cells and osteoclasts in the tibias (Fig. 8a, b), reduced the TRAP5b activity in the serum (Fig. 8c), increased the values of BMD, BV/TV and Tb.Th in the tibias, and decreased the Tb.Sp values of arthritis rats (Fig. 8d). CH223191 nearly completely abolished the above-mentioned effects of tetrandrine and DIM.

## Discussion

Tetrandrine, a member of isoquinoline alkaloids, has been clinically used for the treatment of arthralgia and rheumatic pain in China^9,18^. Our previous studies demonstrated that tetrandrine could modulate the abnormal immune responses and therefore reduce synovitis in CIA mice by restoring the Th17/Treg balance, and attenuate the bone erosion in CIA rats by inhibiting osteoclastogenesis^9,10^. The dual inhibition on the synovitis and bone destruction suggests that tetrandrine might be a more ideal anti-RA drug as compared to the currently-used disease-modifying anti-rheumatic drugs (DMARDs) or anti-osteoclastogenesis agents. The possible target protein and underlying mechanisms of tetrandrine for anti-osteoclastogenesis effect deserve to be identified.

The findings that AhR agonists such as TCDD, Bap and DIM could inhibit osteoclastogenesis^19–21^ and tetrandrine could activate AhR in T lymphocytes^9^ implied that AhR might mediate the anti-osteoclastogenesis effect of tetrandrine. Data of the present study showed that tetrandrine was indeed able to activate AhR in the pre-osteoclasts as evidenced by increasing AhR nuclear import and the expression of CYP1A1, and the anti-osteoclastogenesis effect of either tetrandrine or DIM (a known AhR agonist) was largely diminished by the treatment of CH223191 or AhR siRNA. These results strongly suggested that AhR was the potential target protein of tetrandrine for anti-osteoclastogenesis effect. Of noted, many traditional AhR ligands, especially high-affinity agonists and antagonists of AhR, have significant toxicities, which limit their druggability. The differences in safety and efficacy between tetrandrine and these AhR ligands might result from the diversity of chemical structures and mechanisms of action^22^.

Up to now, little is known regarding the mechanisms by which AhR ligands regulate osteoclastogenesis. Our previous report showed that the inhibitory effect of tetrandrine on the osteoclastogenesis was closely related to the enhancement of the ubiquitination and degradation of Syk. Ohtake F. et al. reported that AhR was a ligand-dependent E3 ubiquitin ligase^23^. These findings reminded us that tetrandrine might promote the ubiquitination and degradation of Syk through AhR. In this study, tetrandrine and DIM were shown to enhance the ubiquitylation and degradation of Syk, while CH223191 and AhR siRNA significantly impaired the action of tetrandrine or DIM, suggesting that the effect of tetrandrine and DIM in promoting the degradation and ubiquitination of Syk was indeed through AhR. On the other hand, the ligands of AhR might function in two distinct ways. The first one is the traditional genomic way, involving the direct expression of many target genes. The second one is the non-genomic way independent of the transcriptional function of AhR, and does not require the participation of Arnt^24^. Tetrandrine and DIM were shown to enhance the degradation of Syk by the way distinct from that to induce the expression of CYP1A1, a well-known genomic way. The findings revealed that tetrandrine and DIM functioned through the non-genomic pathway of AhR.

A new question arises. How do tetrandrine and DIM enhance the ubiquitination of Syk through the non-genomic pathway of AhR? Activated Syk is recognized to be labeled with ubiquitin by E3 ubiquitin ligase, and then multi-ubiquitinated Syk is captured and degraded by the proteasome. E3 ligase c-Cbl has been shown to be involved in the ubiquitination and degradation of Syk in the pre-osteoclasts^25,26^. In this study, c-Cbl siRNA was shown to abolish the effects of tetrandrine and DIM on the ubiquitination and degradation of Syk. Moreover, tetrandrine and DIM enhanced the phosphorylation of c-Cbl in a concentration-dependent manner, which could be diminished by CH223191 or AhR siRNA treatment. These findings revealed that tetrandrine enhanced the degradation of Syk through AhR in a manner dependent on c-Cbl. However, AhR itself is lack of kinase activity. How does it activate c-Cbl? There are reports indicating that the non-receptor tyrosine kinase c-src can bind to AhR in the cytoplasm as a binding protein for AhR. Once AhR is activated by a ligand, c-src is released and activated^27–29^. Furthermore, a large pool of evidence indicated that c-Cbl as the substrate of c-src might be activated^14^. Tetrandrine and DIM could significantly promote the activation of c-src, which was attenuated by the CH223191 or AhR siRNA. Moreover, c-src inhibitor PP2 markedly attenuated the effects of tetrandrine or DIM on the activation of c-Cbl. While, c-Cbl knockdown had no obvious effect on the effect of tetrandrine or DIM for enhancing the activation of c-src. These findings indicated that c-Cbl might be a downstream effector of c-src. Furthermore, we explored the relationships among AhR, c-src, c-Cbl, and Syk. The results obtained from the co-immunoprecipitation assays revealed that tetrandrine might enhance the dissociation of AhR from c-src, and increase the binding of AhR to c-Cbl and Syk. The ubiquitination and degradation of Syk decreased the function of Syk, and then spread to the downstream signaling pathways of Syk^30^. CH223191 treatment significantly diminished the inhibition of tetrandrine or DIM against Syk-PLCγ2 phosphorylation and nuclear translocation of NFATc1.

Finally, in order to verify the above in vitro findings, we investigated the dependence of AhR for tetrandrine and DIM inhibiting osteoclastogenesis and bone destruction in CIA rats. Tetrandrine and DIM were shown to decrease the number of osteoclasts in tibias, and increase the bone mineral density in the tibias. CH223191 diminished the effects of tetrandrine or DIM, indicating that they inhibited osteoclastogenesis and bone destruction in CIA rats through the AhR pathway.

In conclusion, this study identified that tetrandrine enhanced the ubiquitination and degradation of Syk and consequently repressed the osteoclastogenesis and bone destruction through the AhR-c-src-c-Cbl pathway.

## Materials and methods

### Animals

Female Wistar rats (130 g-150 g) were purchased from the Comparative Medicine Center of Yangzhou University (Yangzhou, China). They were maintained in animal center of China Pharmaceutical University with light: dark (12 h: 12 h) condition and fed with commercial diet and water *ad libitum*. The animal experiments were performed with the approval of the guidelines of the Committee on the Care and Use of Laboratory Animals and the related ethical regulations of China Pharmaceutical University.

### Media and reagents

Tetrandrine (C_38_H_42_N_2_O_6_, MW: 622.75, purity ≥ 99%) and DIM (C17H14N2, MW: 246.31, purity ≥ 98%) were purchased from Selleck Chemicals (Houston, USA); RAW264.7 cells were purchased from Fudan IBS Cell Center (Shanghai, China); Dulbecco’s modified Eagle’s media, alpha modification of Eagles medium (α-MEM) and fetal bovine serum (FBS) were purchased from Invitrogen Co. (Carlsbad, CA, USA). Recombinant murine M-CSF was purchased form Bioworld Technology, Inc. (Georgia, USA). Recombinant murine RANKL was obtained from PeproTech Inc. (Rocky Hill, NJ). CH223191 was obtained from MedChem Express, LLC (Princeton, NJ). Special antibodies against p-PLCγ2, PLCγ2, p-Syk, c-src, p-c-src, ubiquitin, Lamin B1, NF-κB-p65 and GAPDH were purchased from Bioworld Technology, Inc. (Georgia, USA). Antibody against Syk was obtained from GeneTex Inc. (Irvine, CA, USA). Antibodies against AhR and Arnt were obtained from Proteintech Inc. (Wuhan, China) Antibodies against NFATc1, CYP1A1, p-c-Cbl and c-Cbl were obtained from ABclonal Techonlogy, Inc. (Wuhan, China). Cycloheximide (CHX) and MG132 were obtained from Braunway Technology Inc. (Beijing, China). Protein A/G agarose was purchased from Shanghai Seven Sea Pharmtech Co., Ltd. (Shanghai, China). TRAP staining kit, complete Freund’s adjuvant and chicken type II collagen (CII) were purchased from Sigma-Aldrich Co., Ltd. (St. Louis, USA). The siRNA targeting AhR (siAhR), siRNA targeting siArnt (siArnt), siRNA targeting sic-Cbl (sic-Cbl) and scrambled RNA (siCtrl) were designed and synthesized by RiboBol Co. (Guangzhou, China). TRAP5b enzyme-linked immunosorbent assay (ELISA) kits were purchased from CusAbio Biotech Co. Ltd. (Wuhan, China)

### Cell culture

Bone marrow-derived macrophages (BMMs), derived from the bone marrow hematopoietic stem cells, were collected from the femurs and tibias of Wistar rats by flushing the marrow space with α-MEM. After removing the red blood cells, the cells were cultured for 1 day in α-MEM containing 10% FBS. The non-adherent cells were collected and cultured with 50 ng/mL M-CSF in α-MEM containing 10% FBS for 4 days, and the adherent cells were used as BMMs. RAW264.7 cells were grown in DMEM supplemented with 10% FBS and maintained at 37 °C in 5% CO_2_ humidified air.

### Osteoclast differentiation

BMMs and RAW264.7 cells were seeded into 48-well plate at a density of 1 × 10^4^ cells per well with a complete medium, RANKL (100 ng/mL) and indicated compounds. The culture medium was replaced every two days. After 5 days, the mature osteoclasts were stained for TRAP using a TRAP staining kit according to the manufacturer’s protocol. The number of TRAP-positive multinucleated cells (nuclei number ≥ 3) was counted under a microscope.

### Gene silencing using siRNA

The experiment was performed according to the protocol reported previously^31^. Briefly, RAW264.7 cells were seeded in 6-well plates 1 day prior to transfection. SiRNAs at a final concentration of 50 nM were transfected into the RAW264.7 cells with gene transfection regent (Cenji Biotech. Inc., Shanghai, China) according to the manufacturer’s instructions. After 48 h, the cells were used for further investigation.

### Western blots analysis

The lysates from cells were prepared by suspending cells in NP40 buffer (Beyotime, Nanjing, China). The same amount of protein lysate was isolated on all 10% SDS-PAGE gels, and then transferred to nitrocellulose filter membranes. The membranes were incubated with primary antibodies overnight at 4 °C after blocked in 5% non-fat milk. After washing, the membranes were incubated with IRDye-conjugated secondary antibody (LI-COR, Inc., Lincoln, MT) for 1 h. After washing, the hybridized bands were got with the help of Odyssey Infrared Imaging System (LI-COR, Inc., Lincoln, MT) [10].

### Co-immunoprecipitation assay

The cells were harvested after washing with ice-cold phosphate-buffered saline, and then lysed in NP40 lysis buffer. The samples were immunoprecipitated with 1 μg of primary antibody against AhR or c-Cbl. After that, 20 μL of protein A/G agarose was blended in the extracted proteins, and rotated for 4 h at 4 °C. The beads were washed with NP40 lysis buffer for three times, and the immunopurified proteins were eluted by and denaturation by boiling the beads in 40 μL of loading buffer.

### Quantitative real-time PCR analysis

Total RNA from cells was isolated using TRIzol reagent (Nanjing SunShine Biotechnology Co., LTD, Nanjing, China) according to the manufacturer’s instructions. Quantitative PCR was performed as previously described^32^. The total mRNAs were reversely transcribed into cDNAs using a cDNA synthesis kit (Abmgoodchina Inc., Zhenjiang, China), and then the cDNAs were amplification by real-time PCR detection system (Bio-Rad, USA) using a SYBR Green PCR kit (Abmgoodchina Inc., Zhenjiang, China). The primer sequences in the reaction used were listed in Table 1. The relative levels of DC-STAMP and TRAP were normalized to the reference gene, GAPDH.

**Table 1:** Primer sequences used in this study

Gene	Forward (F) and reverse (R) primer sequence (5′–3′)	
TRAP	F: ATGACGCCAATGACAAGAGGTTCC	R: TTGTGCCGAGACATTGCCAAGG
DC-STAMP	F: TCTAGTCAGCACTGGCCTCTTCC	R: CTGTTGGCACCTCTCCTCTTCATC
GAPDH	F: TGCCTCTGGTCGTACCACTG	R: GGCAACATAGCACAGCTTCTCT

### Immunofluorescence assay

The immunofluorescence assays for AhR and NFATc1 nuclear translocation were performed just as previously described^33^. The BMMs were fixed in 4% paraformaldehyde, permeabilized with 1% Triton X-100 for 15 min and blocked with 4% bovine serum albumin for 2 h. Next, the cells were incubated with AhR or NFATc1 antibody overnight at 4°C, and next with rhodamine- or FITC-conjugated IgG (Bioworld Technology, Inc., Georgia, USA) for 2 h. Then, the cells were counter-stained with DAPI for 10 min, and photographed using a fluorescence microscope (Olympus, Tokyo, Japan).

### Induction of CIA in Wistar rats and administration of test samples

The Wistar rats were immunized by an intradermal injection at the base of the tail with chicken type II collagen (1.5 mg/mL) emulsified in Freund’s complete adjuvant just as described previously^34^. On day 7, rats were boosted in the same way using the CII emulsified in Freund’s incomplete adjuvant. When the rats showed obvious clinical symptoms (arthritis index score ≥ 3), treatments were started. Tetrandrine, DIM and CH223191, suspended in 0.5% carboxymethylcellulose sodium (CMC-Na), were orally administered for consecutive 14 days. The rats in normal and model groups were orally administered an equivalent volume of 0.5% CMC-Na.

### TRAP staining

At the end of the animal experiment, the left knee joints of rats were obtained and fixed in 4% formaldehyde solution. After decalcified, the joints were embedded in paraffin and sectioned. The sections were stained with a TRAP kit for osteoclast assay.

### Micro-computed tomography (CT) analysis

A micro-CT Scan SkyScan1176 scanner (Bruker Co., Belgium) was used to get the computed tomographic images of the right ankles and knee joints of the rats in all seven groups (*n* = 6) just as described previously^10^. CTvox software (Bruker Co., Belgium) was used to reconstruct the 3-dimensional and 2-dimensional images of the paws and knee joints. The bone mineral density (BMD), trabecular thickness (Tb.Th), trabecular separation (Tb.Sp) and bone volume fraction (BV/TV) of the proximal tibial epiphysis excluding the cortial bone were measured by using a CTan software (Bruker Co., Belgium).

### Serum TRAP5b activity assay

At 1 h after the last administration of the test samples, rats were sacrificed by bleeding, and the sera were prepared. The levels of TRAP5b in the sera were measured using ELISA kits according to the manufacturer’s instructions.

### Statistical analysis

Values were presented as the mean ± S.E.M. Statistically significant differences between the groups were determined by one-way analysis of variance (ANOVA) followed by Tukey’s Multiple Comparison test. At least three independent replicates of each experiment were conducted. Differences were considered statistically significant at *P < 0.05 or **P < 0.01.

## Supplementary information

Supplementary Figures
