# Supplementary material for: Tetrandrine enhances the ubiquitination and degradation of Syk through an AhR-c-src-c-Cbl pathway and consequently inhibits osteoclastogenesis and bone destruction in arthritis
Source: Cell Death Dis. 2019 Jan 15;10(2):38. doi: 10.1038/s41419-018-1286-2 (PMC6427010; doi:10.1038/s41419-018-1286-2)
Supplement: Supplementary file 1 — Supplementary Figures [file 41419_2018_1286_MOESM1_ESM.docx]

**Supplementary Figures**

**
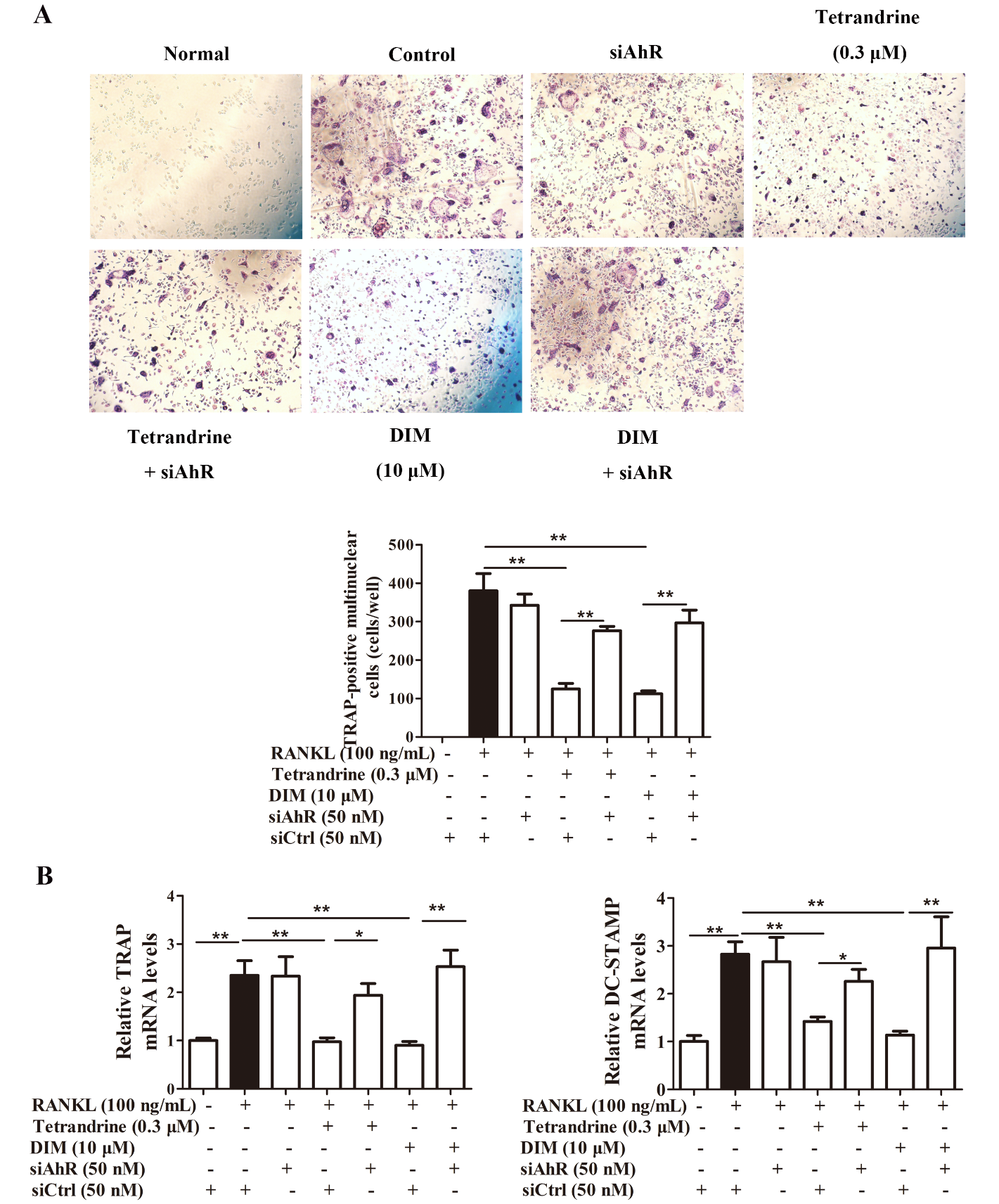
**

**Supplementary Figure 1** **Tetrandrine and DIM inhibited osteoclastogenesis in an AhR-dependent manner.** RAW264.7 cells were transfected with either siAhR or siCtrl. (A) The cells were treated with indicated compounds in the presence or absence of RANKL (100 ng/mL) for 5 days. The osteoclasts were stained using a TRAP kit according to the manufacture’s protocol. TRAP-positive multinucleated cells (nuclei ≥ 3) were counted using an inverted microscope. (B) The cells were treated with indicated compounds in the presence or absence of RANKL (100 ng/mL) for 24 h. The cells were harvested and lysed, and the mRNA expressions of TRAP and DC-STAMP were detected by quantitative PCR. The results are representative of three independent experiments. *^*^P* < 0.05, *^**^P* < 0.01 *vs* indicated group.

**
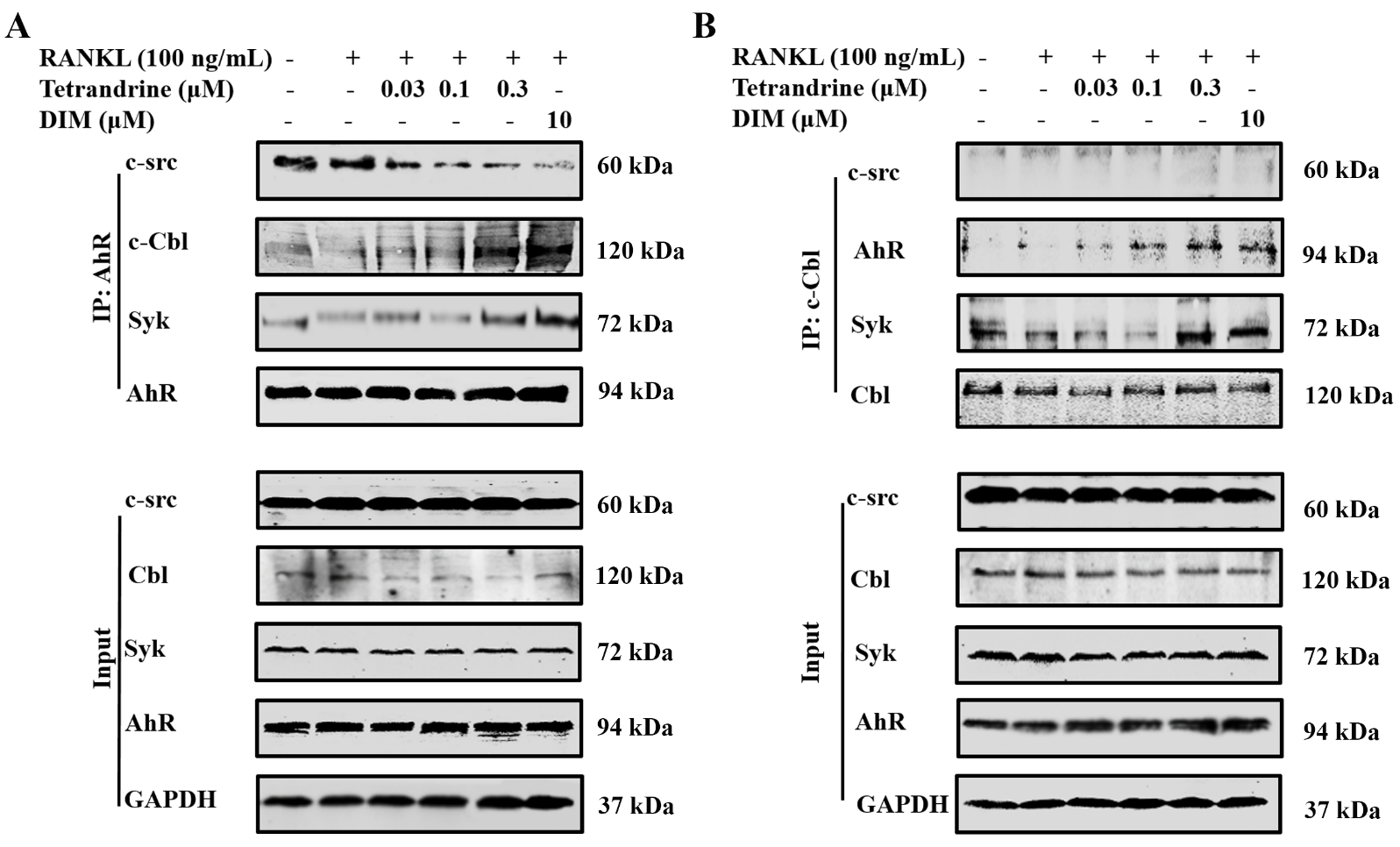
**

**Supplementary Figure 2 Tetrandrine and DIM enhanced the dissociation of AhR from AhR-c-src complex, and the combination with c-Cbl and Syk.** RAW264.7 cells were treated with tetrandrine (0.03, 0.1, 0.3 μM) or DIM (10 μM) for 6 h, and followed with RANKL (100 ng/mL) for 15 min. (A) The proteins were isolated and immunoprecipitated with an antibody against AhR. The levels of c-src, c-Cbl, AhR and Syk in the whole cell lysates and immunoprecipitates were analyzed by western blots. (B) The proteins were isolated and immunoprecipitated with an antibody against c-Cbl. The levels of c-src, c-Cbl, AhR and Syk in the whole cell lysates and immunoprecipitates were analyzed by western blots.
